# Relationship between ferroptosis and mitophagy in cardiac ischemia reperfusion injury: a mini-review

**DOI:** 10.7717/peerj.14952

**Published:** 2023-03-13

**Authors:** Cuihua Liu, Zunjiang Li, Botao Li, Wei Liu, Shizhong Zhang, Kuncheng Qiu, Wei Zhu

**Affiliations:** 1 Third-Grade Pharmacological Laboratory on Traditional Chinese Medicine, State Administration of Traditional Chinese Medicine, Medical College, China Three Gorges University, Yichang, Hubei Province, China; 2 The Second Clinical College of Guangzhou University of Chinese Medicine, Guangzhou University of Chinese Medicine, Guangzhou, Guangdong Province, China

**Keywords:** Ferroptosis, Mitophagy, Relationship, Myocardial ischemia reperfusion injury, Mechanism

## Abstract

Cardiovascular diseases (CVD), with high morbidity and mortality, seriously affect people’s life and social development. Clinically, reperfusion therapy is typically used to treat ischemic cardiomyopathy, such as severe coronary heart disease and acute myocardial infarction. However, reperfusion therapy can lead to myocardial ischemia reperfusion injury (MIRI), which can affect the prognosis of patients. Studying the mechanisms of MIRI can help us improve the treatment of MIRI. The pathological process of MIRI involves many mechanisms such as ferroptosis and mitophagy. Ferroptosis can exacerbate MIRI, and regulation of mitophagy can alleviate MIRI. Both ferroptosis and mitophagy are closely related to ROS, but there is no clear understanding of the relationship between ferroptosis and mitophagy. In this review, we analyzed the relationship between ferroptosis and mitophagy according to the role of mTOR, NLPR3 and HIF. In addition, simultaneous regulation of mitophagy and ferroptosis may be superior to single therapy for MIRI. We summarized potential drugs that can regulate mitophagy and/or ferroptosis, hoping to provide reference for the development of drugs and methods for MIRI treatment.

## Introduction

Cardiovascular diseases (CVD), including hypertension, coronary heart disease and myocardial infarction, not only pose a serious threat to human health but also bring heavy economic burden to patients (Andersson & Vasan, 2018; Geraghty et al., 2021). Coronary heart disease, myocardial infarction and other ischemic cardiomyopathy are mostly caused by long-term ischemia of myocardial tissue. Timely reperfusion therapy is an effective clinical treatment method at present. However, myocardial ischemia reperfusion injury (MIRI) caused by reperfusion therapy can affect the prognosis of patients (Hausenloy & Yellon, 2013; Zhao et al., 2022). Since MIRI cannot currently be treated effectively, studying its pathological mechanism is crucial to improving CVD treatment.

As we know, mitochondria are extremely important since the heart needs efficient oxidative metabolism (Kumar, Kelly & Chirinos, 2019; Pohjoismaki & Goffart, 2017). Mitochondrial dysfunction will lead to ROS overproduction, which is considered to be the critical cause of MIRI (Jiang et al., 2021). In cardiomyocytes, mitochondria maintain quantity, quality and basic functions through mitophagy which is a kind of selective autophagy (Yang et al., 2019). Mitophagy can degrade damaged mitochondria and reduce ROS production, so it is of great significance for maintaining normal physiological functions of the heart.

Ferroptosis, a new form of cell death is identified as a primary mechanism of MIRI (Gao et al., 2015). Ferroptosis inhibition is emerging as an effective method to treat MIRI (Stamenkovic et al., 2021; Zhang et al., 2021a). At present, the understanding of ferroptosis is still limited. It is already known that the pathological process of ferroptosis is not only closely related to ROS (Li et al., 2020a; Park & Chung, 2019; Su et al., 2019) but also closely related to mitochondrial dysfunction (Gan, 2021; Gao et al., 2019b; Sumneang et al., 2020). Taking morphology into consideration, the ultrastructure of mitochondria can be affected by ferroptosis such as volume reduction, increased bilayer membrane density, outer mitochondrial membrane disruption and so on (Battaglia et al., 2020). It is known that mitophagy can degrade damaged mitochondria and thus inhibit mitochondrial dysfunction, but the relationship between mitophagy and ferroptosis remains unclear. We thus speculate that there may be some direct or indirect relationship between mitophagy and ferroptosis in MIRI, and analyzing the mechanisms of ferroptosis and mitophagy may provide some valuable clues.

Although with advanced development of drug management, CVD remain the most common cause of death worldwide (Liu et al., 2019a; Townsend et al., 2022), which result in a high burden of comorbidities to physicians in clinic. Thus, newly and developed drug management of CVD aimed for special mechanism remains challenging and urgently explored. In this study, we hypothesized that elaborating the relationship between mitophagy and ferroptosis would help us find effective treatments for MIRI. We intended to investigate and review the main mechanisms that lead to ferroptosis by exploring the relationship between mitophagy and ferroptosis involved in CVD progress. In addition, we also summarized the potential drugs including natural compounds and drugs used in alternative medicine that could alleviate MIRI *via* regulating mitophagy and/or inhibiting ferroptosis, providing reference for the treatment of MIRI. This study was a mechanism-oriented review that explored the innovative relationship between mitophagy and ferroptosis participated in the development of CVD, which could provide new insight for CVD treatment and bring hope to patients by improving clinical efficacy, improving patient prognosis and increasing the quality of life of CVD patients.

## Survey Methodology

Literature searches were conducted in the PubMed, Web of Science and Chinese National Knowledge Infrastructure databases. In addition to articles published since 2017, earlier articles were also considered. The keywords used were as follows: myocardial ischemia reperfusion injury, ferroptosis, mitophagy, myocardial ischemia reperfusion injury and ferroptosis, mitophagy and myocardial ischemia reperfusion injury, iron metabolism, the mechanism of ferroptosis, the mechanism of mitophagy. As our work gradually unfolded, we then searched literature by keywords HIF and mitophagy, HIF and ferroptosis, NLRP3 and mitophagy, NLRP3 and ferroptosis, mTOR and mitophagy, mTOR and ferroptosis, natural compounds with mitophagy and/or ferroptosis. After removing duplicate articles and the articles with little relevance, 151 articles were selected for this review.

### MIRI and mitophagy

Because of the heart’s high demand for energy, normal mitochondrial function is essential for heart development (Pohjoismaki & Goffart, 2017; Zhang et al., 2020a). MIRI is often accompanied by mitochondrial damage and dysfunction in cardiomyocytes. Assuring myocardial cells have enough mitochondria to fulfill their physiological needs, a variety of quality control mechanisms have evolved in mitochondria including mitophagy, biogenesis, mitochondrial dynamics, *etc.* (Li et al., 2021a). In 2005, Lemasters proposed “mitophagy” firstly to emphasize the non-random nature of the mitochondrial selective autophagy process (Lemasters, 2005). Mitophagy is the main mechanism for maintaining mitochondrial homeostasis in cardiomyocytes by degrading the dysfunctional mitochondria.

The occurrence of MIRI will go through two stages: ischemia and reperfusion. In the myocardial ischemia stage, hypoxia environments caused by ischemia can affect the process of oxidative phosphorylation of mitochondria, resulting in insufficient myocardial energy supply (Killackey, Philpott & Girardin, 2020). The lack of energy activates the AMPK pathway, and then mitophagy is activated (Kim et al., 2011; Laker et al., 2017). Activated mitophagy is mainly used to degrade aging mitochondria to cope with the energy crisis. Meanwhile, ROS accumulation is gradually induced by hypoxia at this stage. As ischemia and hypoxia continue, the lack of energy leads to the inability of Ca^2+^ to be excreted by the calcium pump, resulting in the accumulation of Ca^2+^ in cardiac myocytes. In order to maintain Ca^2+^ homeostasis in cardiomyocytes, mitochondria will absorb excessive Ca^2+^ from cytoplasm, resulting in Ca^2+^ overload in mitochondria. Ca^2+^ overload (Kinnally et al., 2011) and ROS accumulation (Leucker et al., 2011; Pravdic et al., 2009) lead to the mitochondrial mPTP opening, then mitochondrial membrane potential collapse (Zorov, Juhaszova & Sollott, 2014). In the reperfusion stage, the restoration of oxygen supply leads to ROS burst, which in turn prolongs the opening time of mitochondrial mPTP, further damaging the mitochondria. The ROS released by damaged mitochondria induces more ROS generation (Zorov et al., 2000), creating a vicious cycle. At this point, mitophagy, which can reduce the production of ROS by degrading the damaged mitochondria, is very important for MIRI mitigation. It should be noted that although most studies have shown that promoting mitophagy can alleviate MIRI, some studies have also shown that excessive mitophagy also damages cardiac myocytes, and inhibition of mitophagy is required at this time (Huang et al., 2022; Wu et al., 2020).

### Ferroptosis and MIRI

Ferroptosis is a new type of programmed cell death that was first discovered by Dolma in 2003 and named by Dixon in 2012 (Dolma et al., 2003; Dixon et al., 2012). Programmed cell death, such as apoptosis, necrosis, and other forms, clears out damaged or infected cells, allowing surrounding healthy cells to perform their functions better (D’Arcy, 2019). Unlike reported forms of programmed cell death, ferroptosis is an iron-dependent form of cell death that is accompanied by massive iron accumulation and lipid peroxidation (Li et al., 2020a). Nowadays, Ferroptosis has been shown to exist in the pathological process of a variety of diseases, such as in cancers (Qiu et al., 2022; Xu et al., 2019), brain diseases (Weiland et al., 2019), kidney diseases (Tang & Xiao, 2020), MIRI (Zhao et al., 2021) and other diseases. For some ferroptosis-susceptible tumors, activation of ferroptosis is a potential treatment strategy (Yu et al., 2017). But in cardiac tissue, ferroptosis which can lead to cardiomyopathy needs to be suppressed (Fang et al., 2019).

The relationship between ferroptosis and MIRI was first revealed by Gao et al. (2015). Inhibition of ferroptosis *via* inhibiting glutaminolysis can protect heart tissue from MIRI *in vitro* heart model. Inhibition of myocardial ferroptosis can also alleviate MIRI in diabetic rats by inhibiting endoplasmic reticulum stress (Li et al., 2020b). According to recent studies, ferroptosis which is iron-dependent is accompanied by lipid peroxide (LPO) accumulation (Dixon et al., 2012; Stamenkovic, Pierce & Ravandi, 2019). Ferroptosis inhibitor Fer-1 has been reported can inhibit peroxidation, and prevent the accumulation of LPO thereby inhibiting ferroptosis and subsequently alleviating MIRI (Dixon et al., 2012; Li et al., 2019; Miotto et al., 2020). Fer-1 can also protect the heart from cardiomyopathy by maintaining mitochondrial function (Fang et al., 2019). Fang et al. found another ferroptosis inhibitor Lip-1 could reduce myocardial infarct sizes and maintain mitochondrial structure and function to prevent MIRI (Feng et al., 2019). In addition, on the outer mitochondria membrane, ischemia and other pathological stimuli can be protected against by the mammalian target of rapamycin (mTOR). When mTOR is overexpressed, erastin (a ferroptosis inducer) induced cell death is inhibited, while mTOR deletion will exaggerate the cell death (Baba et al., 2018). According to these data, mitochondria are important for ferroptosis-induced cardiomyocyte death. At present, the main systems for inhibiting ferroptosis include: the Cyst (e)ine/GSH/GPX4 Axis, the NAD (P)H/FSP1/CoQ10 System, and the GCH1/BH4/DHFR System (Zheng & Conrad, 2020), but the research focus is mainly on iron homeostasis, system Xc- and GPX4 (Li et al., 2021b; Cao & Dixon, 2016).

### Absorption and utilization of iron

Iron homeostasis is a vital element for fundamental biological functions of human body, accumulating evidences have shown that iron dyshomeostasis is involved in the pathogenesis of cardiovascular diseases (Wei et al., 2022). When iron homeostasis is disrupted because of iron deficiency or overload, it can lead to rapid lipid peroxidation of cells due to lack of GPX4 (Ouyang et al., 2021), resulting in cardiovascular cellular damage and accelerating the occurrence of various diseases including atherosclerosis, MIRI, coronary heart disease and so on (Gao et al., 2019a; Kobayashi et al., 2018). Until now, regulating iron acquisition, recycling, and storage is the main method of controlling system iron levels for human (Wallace, 2016), which is mainly by unidirectional recycling of iron from senescent red blood cells to the erythroid bone marrow through macrophages, the cycling of iron from hepatocytes to the blood and vice versa, and iron absorption through duodenal and upper Jejunum (Piperno, Pelucchi & Mariani, 2020).

**Figure 1:**
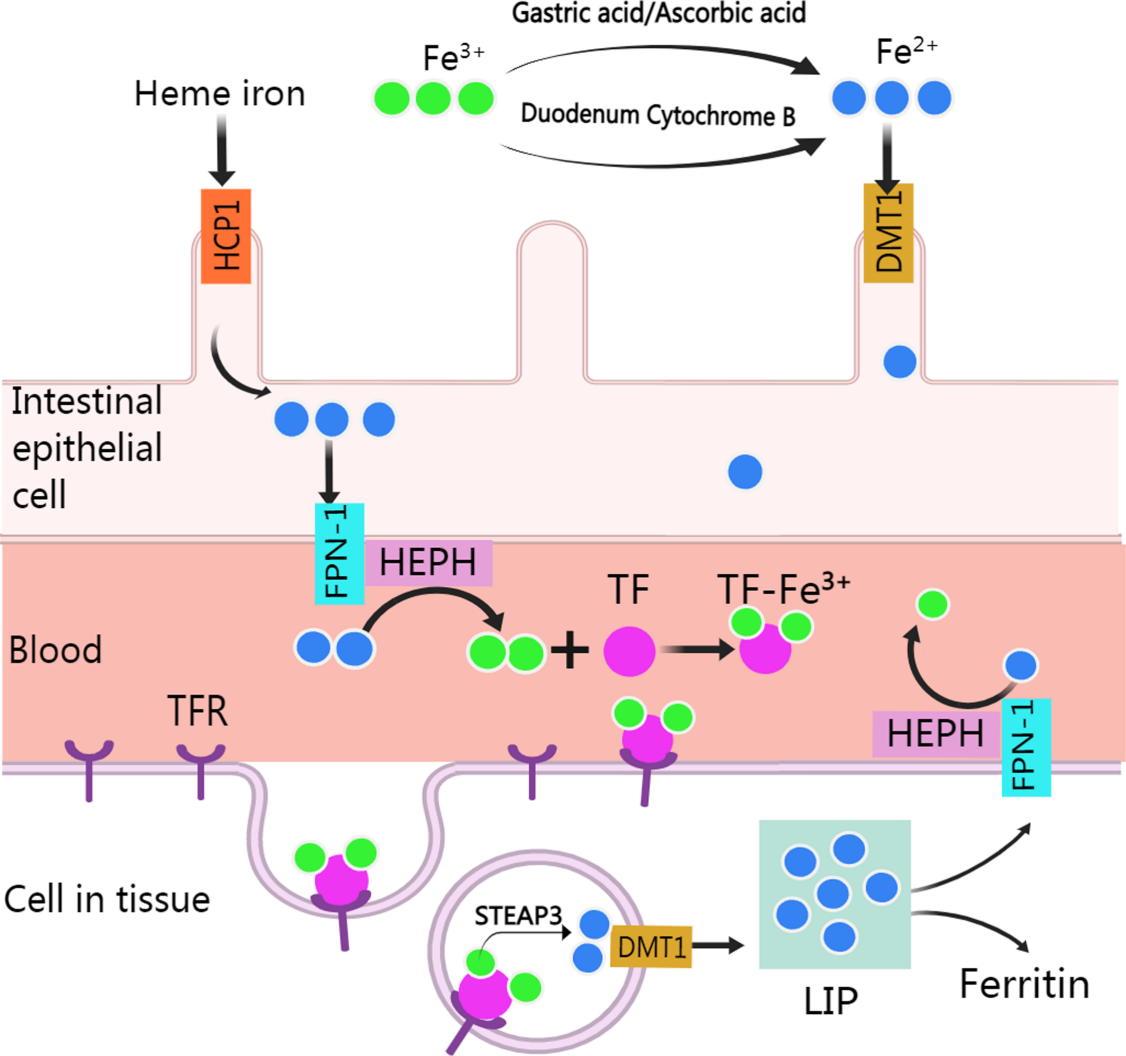
Absorption and utilization of iron. Created with MedPeer: http://image.medpeer.cn.

Normally, human body absorbs iron from food or other nutritious in the type of heme and non-heme (or inorganic) forms except a small amounts of iron are lost through skin exfoliation, gastrointestinal exfoliation, and urine and bile excretion (Gulec, Anderson & Collins, 2014). After entering the body from food, iron experience various metabolic processes before it can be used (Fig. 1). Heme iron (Fe^2+^) can be absorbed directly *via* heme/folate transporter 1 at the apical membrane of intestinal epithelial cells (West & Oates, 2008; Zhang et al., 2019a). Non-heme iron (Fe^3+^) in food is partly reduced and dissolved by gastric acid and ascorbic acid, and the rest is reduced to Fe^2+^ by cytochrome B, which is then transported to intestinal epithelial cells by divalent metal-ion transporter 1 (DMT 1, encoded by the SLC11A2 gene) for absorption. Subsequently, after traversing the basolateral membrane *via* ferroportin 1, Fe^2+^ is oxidized to Fe^3+^ by Hephaestin (HEPH), and then binds with transferrin (TF) to form TF-Fe^3+^ complex for utilization by organs (Gulec, Anderson & Collins, 2014). After the TF-Fe^3+^ complex binds with the transferrin receptor (TFR) and enters the endosome through endocytosis, Fe^3+^ is released from the TF-Fe^3+^ complex, then is reduced to Fe^2+^ by STEAP3 and crosses the endosomal membrane into the cytoplasm by DMT (Sendamarai et al., 2008). The imported Fe^2+^ enters a metabolically cytosolic labile iron pool, which is used for incorporation into prosthetic groups of iron-dependent enzymes and proteins, incorporation into heme and iron-sulfur cluster biogenesis, and storage in ferritin. The excess iron is exported back to the circulation by ferroportin 1, during which Fe^2+^ is oxidized to Fe^3+^ by HEPH in plasma and recombined with TF (Manz et al., 2016).

## Mechanism of ferroptosis

### Iron overload

Iron overload is an important factor in activating ferroptosis. Iron overload usually occurs from a genetic disease or iatrogenic (Godbold & McFarland, 2021; Murphy & Oudit, 2010; Piperno, Pelucchi & Mariani, 2020). In pathological conditions caused by some diseases, iron overload can result from increased iron intake, increased gastrointestinal absorption (Godbold & McFarland, 2021), and accumulation of non-heme iron through heme degradation (Fang et al., 2019), *etc*. When the body is in a pathological condition of iron overload, the capacity of plasma transferrin to bind iron is saturated, leading to the accumulation of non-transferrin bound iron (Brissot et al., 2012). The accumulation of non-transferrin bound iron in plasma accelerates the deposition of iron in tissues, particularly excitable tissues containing Ca^2+^ channels which are known to conduct Fe^2+^ into cells (Zhang et al., 2019a). Therefore, iron overload may be an important cause of MIRI-induced cardiac ferroptosis, because cardiac tissue contains high levels of functional voltage-gated Ca^2+^ channels.

Iron overload is often accompanied by the imbalance of iron storage and release in our bodies. Ferritin, including ferritin heavy chain 1, ferritin light chain, as well as TFR, is currently the focus of ferroptosis research due to its ability to store iron (Li et al., 2022a; Zhang et al., 2021b). Ferroptosis inducer RSL3 increased iron uptake by upregulating the expression of TFR, while down-regulating the expression of ferritin heavy chain 1 and ferritin light chain, reducing iron storage, leading to the release of large amounts of free iron and thus inducing ferroptosis (Yang & Stockwell, 2008). Additionally, hypoxia inducible factor 1 (HIF-1) and iron regulatory protein (IRP, also known as IREB) have also been reported can increase the expression of TFR and increase iron uptake (Cheng et al., 2015; Tacchini et al., 1999; Torti & Torti, 2013). Tang et al. (2008) further found that HIF-1 *α* activation not only induced TFR expression increasing, but also increased transferrin uptake and iron accumulation, exacerbated oxidative damage that increased the lipid peroxidation. Therefore, inhibition of ferritin (Torti & Torti, 2013) or ferritin deficiency (Fang et al., 2020) can induce ferroptosis.

The ferroptosis during MIRI is closely related to mitochondrial ROS. When ferritin is not expressed enough, excessive intracellular free iron will cause oxidative stress and impaired mitochondrial function in the heart, manifested by decreased mitochondrial respiration, depolarization of mitochondrial membrane potential, and mitochondrial swelling (Sumneang et al., 2020). The mitochondrial dysfunction leads to a large number of ROS production, mainly including O_2^−^_ andH_2_O_2_, which can promote the Fenton reaction. Fenton reaction consists of three reactions, Fe^2+^ + H_2_O_2_ → Fe^3+^ + OH^−^ + OH•, H_2_O_2_ + 2Fe^3+^ → 2Fe^2+^ + O_2_ + 2H^+^ and O_2^+^_ Fe^2+^ → Fe^3+^ + O_2^−^_. We know that H_2_O_2_, OH•, O_2^−^_ all belong to ROS. The continuous production of ROS by Fenton reaction further damages the mitochondria and leads to more ROS generation, creating a vicious cycle. In addition, the accumulating ROS will damage cellular proteins, lipids and DNA, causing cell and tissue damage and eventually lead to ferroptosis (Valko et al., 2016). In a limited number of studies, cardiac ferroptosis has also been studied. However, the mechanistic association between cardiac ferroptosis and iron overload needs further investigation (Sumneang et al., 2020).

### Lipid peroxidation

**Figure 2:**
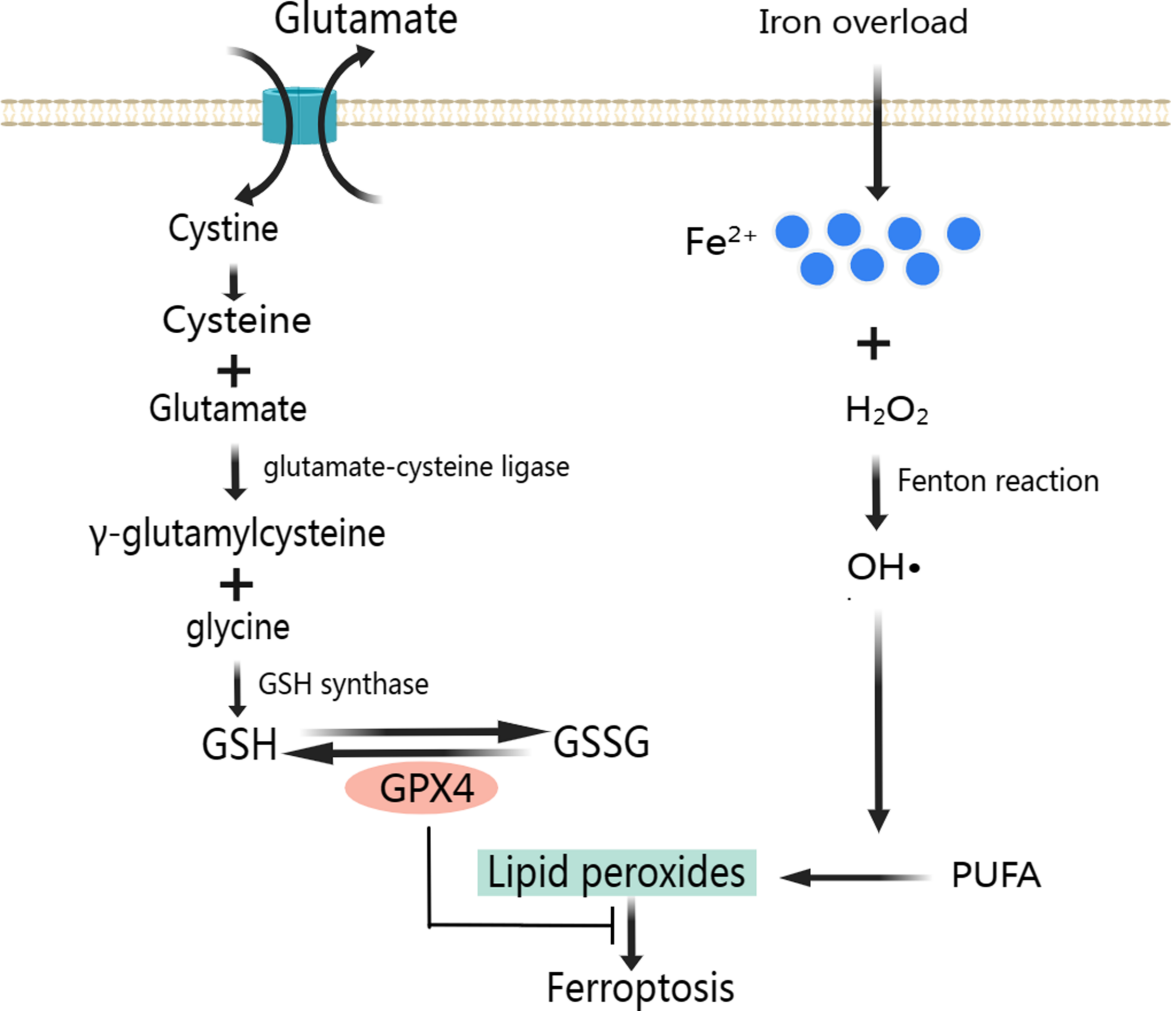
Classic mechanism of ferroptosis. Created with MedPeer: http://image.medpeer.cn.

A large number of ROS produced mainly through Fenton reaction continues to participate in lipid peroxidation (Fig. 2). Lipid peroxidation has been implicated in almost all human diseases associated with oxidative stress of cause in MIRI, and it has been used to assess the degree of ferroptosis (Chen et al., 2021). Attenuating lipid peroxidation can inhibit ferroptosis and thus alleviates CVD (Bai et al., 2020; Tadokoro et al., 2020).

As a result of lipid peroxidation, polyunsaturated fatty acids (PUFA) and phosphatidylethanolamine (PE) are oxidatively decomposed. PUFA is the main component of phospholipids in cell and organelle membranes, it is also an important substrate for the synthesis of PE, the main component in the inner layer of the phospholipid bilayer. PUFA has a high affinity with ROS. ROS of hydroxyl radicals (OH•) and hydrogen peroxide (H_2_O_2_) first acquire hydrogen atoms in PUFA to produce Lipid ROS (L-). Then, the Lipid radicals react with the oxygen molecule to form Lipid peroxyl radicals (LOO-). Lipid peroxyl radicals extract hydrogen atoms from other PUFA to form a new LOO- and Lipid hydroperoxide (LOOH). LOO- can continuously react with PUFAs, which makes the lipid peroxidation of PUFAs have the characteristics of cascade reaction (Ma et al., 2021). PUFA, arachidonic acid and adrenal acid are synthesized into poly-unsaturated fatty acid-phosphatidyl ethanolamine (PUFA-PE). With the participation of Lipoxygenase, PUFA-PE underwent lipid peroxidation reaction in the plasma membrane and endoplasmic reticulum, and finally formed LPO (Wang & Li, 2019). The lipid peroxidation reaction of ROS with PUFA and PE destroys the fluidity and stability of cell membrane, increases the permeability of cell membrane, and eventually leads to cell death.

### GPX4 and system Xc- in preventing ferroptosis

To prevent ferroptosis, oxidative stress caused by LPO needs to be inhibited, and GPX4 is the key regulator in this process (Fig. 2) (Park et al., 2019). GPX4 is a unique intracellular antioxidant enzyme that can directly reduce LPO production in cell membranes to non-toxic lipid alcohols (Imai et al., 2017; Ursini & Maiorino, 2020). Under the catalytic action of GPX4, H_2_O_2_ and LPO were reduced, and GSH was oxidized to disulfide-oxidized form (GSSG) (Lu, 2013). When GSH is depleted, GPX4 will be inactivated leading to LPO accumulation and ultimately ferroptosis (Xie et al., 2016; Yang et al., 2014).

GSH is composed of glycine, glutamate and cysteine. And cysteine uptake depends on the activation of cystine/glutamate reverse transport system Xc-. System Xc- is a heterodimeric cell surface amino acid antiporter composed of two subunits, a light-chain subunit SLC7A11 (xCT) and a heavy-chain subunit SLC3A2 (CD98, 4F2hc), which are linked by an extracellular covalent disulfide bond and play different roles. System Xc- imports extracellular cystine in exchange for intracellular glutamate at a ratio of 1:1 (Cao & Dixon, 2016; Liu, Zhu & Pei, 2021). Intracellular cystine is first reduced to cysteine, then cysteine and glutamate form *γ*-glutamylcysteine under the catalysis of glutamate-cysteine ligase. Next, GSH is formed from *γ*-glutamylcysteine and glycine under the catalysis of GSH synthase. Also, GSH can regulate glutamate-cysteine ligase through negative feedback (Lu, 2013).

When system Xc- dysfunction occurs, the cell redox becomes unbalanced. A study of glioma cells showed that knockdown of SLC7A11 increased ROS production and decreased glutathione production, resulting in increased cell death under oxidative and genotoxic stress, and overexpression of SLC7A11 leads to increased resistance to oxidative stress (Polewski et al., 2016). The system Xc- can be inhibited irreversibly by ferroptosis inducer erastin (Sato et al., 2018). In cancer cells, silencing SLC7A11 makes them more sensitive to ferroptosis induced by erastin, while overexpressing SLC7A11 makes them more resistant to it (Dixon et al., 2012). In addition, the tumor suppressor gene P53 can inhibit the expression of SLC7A11, and then inhibit the uptake of cystine by cells, ultimately leading to cell ferroptosis (Jiang et al., 2015). Overexpressing SLC7A11 in cardiomyocytes can restore cardiac GSH and cystine levels and reduce ferroptosis (Fang et al., 2020). In summary, GPX4 and system Xc- are both key regulators of ferroptosis.

### Relationship between ferroptosis and mitophagy in MIRI

**Figure 3:**
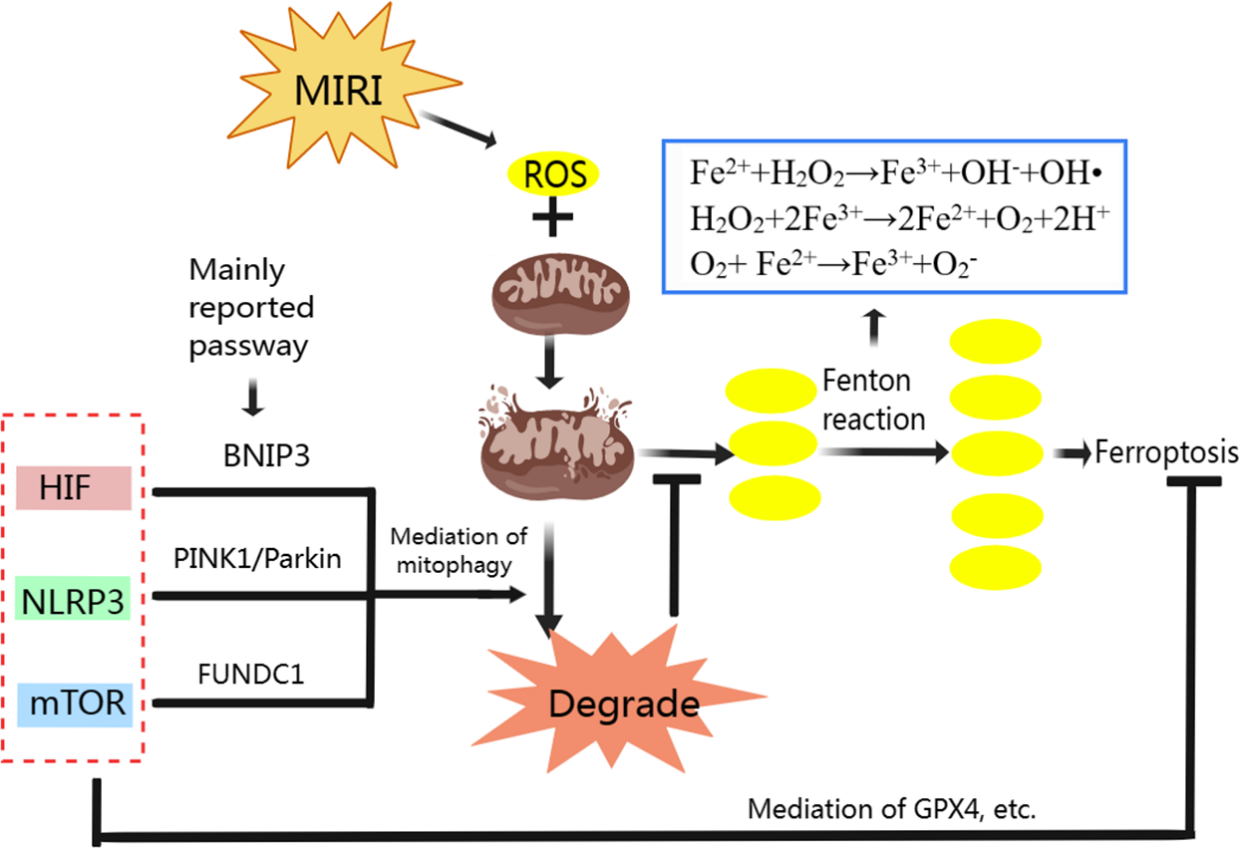
Regulation of ferroptosis and mitophagy by HIF, NLRP3 and mTOR. Created with MedPeer: http://image.medpeer.cn.

We have analyzed the relationship between mitophagy and MIRI as well ferroptosis and MIRI respectively, and the mechanism of ferroptosis. Mitophagy also has complex regulatory mechanisms. Classic mitophagy pathways include PINK1/Parkin, BNIP3/Nix and FUNDC1 pathway (Qiu et al., 2021). Mitophagy and ferroptosis are closely related to ROS during MIRI’s pathological process (Fig. 3). Moderate mitophagy can degrade damaged mitochondria and reduce excessive ROS production (Yang et al., 2020), whereas ferroptosis is accompanied by a large amount of ROS production and ultimately leads to myocardial cell death (Nakamura, Naguro & Ichijo, 2019; Su et al., 2019). It is an interesting question whether regulating mitophagy and reducing mitochondrial ROS production can inhibit ferroptosis in MIRI. From the perspective of mechanism, we found that HIF-1, mTOR and NLRP3 all play important roles in regulating mitophagy and ferroptosis, acting as “messengers”.

#### HIF in ferroptosis and mitophagy

HIF-1, which has three subtypes in mammals including HIF-1 *α*, HIF-2 *α*, and HIF-3 *α*, is a heterodimer transcription factor that plays a key role in mediating adaptive responses to hypoxia. HIF is closely associated with ferroptosis. In the hypoxic environment, HIF-1 is involved in the increase of TFR gene transcription in Hep3B human hepatoma cells (Tacchini et al., 1999). In colorectal cancer, activation of HIF-2 *α* potentiates oxidative cell death by increasing cellular iron (Singhal et al., 2021). In mouse testis, accumulation and stabilization of HIF-1 *α* induced by a widely used plasticizer (di (2-ethylhexyl) phthalate, DEHP) lead to ferroptosis in Leydig and Sertoli cells (Wu et al., 2022a). In the MIRI model, HIF-1 *α* has also been reported to induce TFR expression and iron absorption, exacerbate cellular oxidative damage and increase lipid peroxidation (Tang et al., 2008). These studies suggest that HIF especially HIF-1 *α* overexpression can induce ferroptosis mainly through storage, absorption and accumulation of iron.

HIF also plays important role in maintaining normal mitochondrial function. The HIF-1 *α* could improve mitochondrial function, decrease cellular oxidative stress, activate cardio-protective signaling pathways (Zheng et al., 2021). When MIRI occurs, promoting the expression of HIF-1 *α* and BNIP3 can promote BNIP3-mediated mitophagy, thus alleviating MIRI (Liu et al., 2019b; Zhang et al., 2019b; Zhu et al., 2020). BNIP3 and Nix (BNIP3L, homologous protein of BNIP3) are proteins on the surface of the mitochondrial membrane (Dorn, 2010). When mitophagy is activated, the LC3-interacting region (LIR) on BNIP3 and Nix can bind to LC3 on the membrane of the autophagosome, promoting the combination of damaged mitochondria with the autophagosome to complete mitophagy (Marinkovic, Sprung & Novak, 2021). Specifically, in the ischemia stage of MIRI, BNIP3 acts as a mitochondrial sensor of oxidative stress (Kubli et al., 2008) and HIF-1 initiates mitophagy mainly by activating BNIP3 and Nix (Martinez Vicente, 2017). In the reperfusion stage, BNIP3 is further activated due to ROS outburst, which promotes the initiation of mitophagy (Kubli et al., 2008; Ma et al., 2012).

Although both ferroptosis and mitophagy can be regulated by HIF. Paradoxically, increased HIF expression induces ferroptosis to aggravate MIRI, and increased HIF expression promotes mitophagy to alleviate MIRI. Some researchers inhibited GPX4 activity with ferroptosis inducers, and the production of lipid peroxides in the cells began to increase, followed by mitochondrial damage, mitochondrial ROS increased, and eventually lead to cell ferroptosis. When mitochondria targeted ROS scavenger agent (MitoQ) was used, mitochondrial morphology and function were preserved and cell death was prevented, despite the GPX4 inhibition and lipid peroxidation remained (Jelinek et al., 2018). Therefore, we suggest that ferroptosis in MIRI and even in ischemic cardiomyopathy may be mainly mitochondrial ROS dependent. Appropriate mitophagy can degrade damaged mitochondria and reduce ROS production, and the reduction of mitochondrial ROS makes Fenton reaction lack the necessary substrate H_2_O_2_, so even if HIF expression is increased, ferroptosis is not actually promoted.

#### NLRP3 in ferroptosis and mitophagy

NLRP3 is strongly associated with ferroptosis due to the level change of ROS. The NLRP3 inflammasome is a critical component of the innate immune system and inflammation, mediating caspase-1 activation and secretion of the pro-inflammatory cytokine IL-1 *β*/IL-18 in response to numerous danger signals and pathogens (Kelley et al., 2019). In a swine MIRI model (cardiac arrest followed by resuscitation), the NLRP3 expression, IL-1 *β* and IL-18 contents, iron deposition in myocardial tissue were significantly increased, while the GPX4 expression was significantly decreased (Xu et al., 2021a). In an H9C2 cell model induced by hyperglycemia, hypoxia, and reoxygenation, NLRP3 protein expression increased, GPX4 expression decreased, and ferroptosis increased under the influence of ROS (Wang et al., 2020a). Therefore, the effect of NLRP3 on ferroptosis is mainly through the level of ROS, in which GPX4 plays an important role in reducing ROS and inhibiting ferroptosis.

NLRP3 is also associated with CVD, including atherosclerosis, MIRI, heart failure, *etc*. (Wang et al., 2020b). The ROS is reported can promote NLRP3 production, and NLRP3 can also be released to promote ROS production (Wang et al., 2021a). Mitophagy can eliminate damaged mitochondria and reduce ROS production, thereby inhibiting NLRP3 inflammasome activation (Kim, Yoon & Ryu, 2016; Mangan et al., 2018; Wu & Cheng, 2022). Therefore, NLRP3 is one of the important therapeutic targets to alleviate MIRI. And NLRP3-related mitophagy relies on the activation of the mitophagy pathway of PINK/Parkin which plays a crucial role in cardiovascular disease prevention and treatment (Wu et al., 2022b). In normal mitochondria, the serine/threonine kinase PINK1 is transferred to the mitochondrial inner membrane for degradation. When mitochondria are damaged, such as membrane depolarization, mitochondrial complex dysfunction, mutagenic stress, *etc*., PINK1 accumulates on the outer membrane of injured mitochondria, recruiting Parkin from the cytoplasm to the mitochondrial and activating it. Activated Parkin ubiquitinated mitochondrial membrane proteins allow mitochondria to be recognized and swallowed by autophagic vesicles, and eventually fuse with lysosomes and be degraded (Ji et al., 2021; Li et al., 2021c). NLRP3 inflammasome activation can be reduced by PINK1-mediated mitophagy during cerebral and hepatic ischemia-reperfusion injury (He et al., 2019; Xu et al., 2020). Although it has not been reported so far, we speculate that PINK1-mediated mitophagy plays an important role in NLRP3 inhibition and thus alleviates MIRI.

#### mTOR in mitophagy and ferroptosis

As a serine-threonine kinase, mTOR, which is made up of mTORC1 and mTORC2, is an essential controller in cell growth and metabolism. In addition, mTOR is also involved in ferroptosis regulation. In tumor cells, mTOR inhibition can lead to GPX4 degradation and promotes ferroptosis (Liu et al., 2021a). When mTOR is inhibited in cardiomyocytes, cellular iron accumulates, resulting in iron overload (Bayeva et al., 2012). Overexpression of mTOR can prevent ferroptosis by suppressing ROS production (Baba et al., 2018), but mTOR overexpression also inhibits mitophagy (Wang et al., 2021b; Zhang et al., 2020b). Yan Xiao, et al. found that electroacupuncture pretreatment could increase the expression of mTOR and down-regulate the expression of FUNDC1 to inhibit mitophagy and thereby alleviate MIRI (Xiao et al., 2020). The FUNDC1 protein is important in CVD because it is the receptor for hypoxia-induced mitophagy on mitochondrial membranes (Li et al., 2021a; Liu, Li & Chen, 2021; Wu et al., 2016). In normal conditions, FUNDC1 exists stably in mitochondria’s outer membrane. When mitochondria are damaged or dysfunctional, FUNDC1 is dephosphorylated under the action of related enzymes, and the interaction between its LIR domain and LC3 is enhanced, activating mitophagy (Liu et al., 2012). Controversially, promoting and/or inhibiting mitophagy to alleviate MIRI *via* FUNDC1 both have been reported (Dong et al., 2022; Liu, Li & Chen, 2021). This involves the question that whether mitophagy was excessive. In MIRI, due to the influence of various factors such as ischemia and reperfusion time, appropriate promotion of mitophagy with inhibiting mTOR expression can degrade damaged mitochondria, reduce ROS and relieve MIRI, but excessive mitophagy will lead to apoptosis of cardiac myocytes (Cao et al., 2019; Qiu et al., 2018). Sometimes, excessive mitophagy is also accompanied by excessive autophagy, which leads to ferritin degradation, ROS accumulation and ultimately ferroptosis (Hou et al., 2016; Zhu et al., 2019). Therefore, mTOR overexpression is beneficial only when mitophagy is excessive, by inhibiting both mitophagy and ferroptosis during MIRI. And in the early stages of MIRI, hypoxia and lack of energy can inhibit mTOR and activate mitophagy but inhibition of mTOR can promote ferroptosis. If our previous hypothesis is true that ferroptosis in MIRI is mainly mitochondria ROS dependent, although the expression of mTOR is suppressed, the ferroptosis may not be promoted because the activation of mitophagy reduces ROS production by inhibiting the Fenton reaction and thus inhibiting ferroptosis. Unfortunately, there is no clear standard to judge whether mitophagy is excessive in the existing MIRI animal and cell models. We think that the role of mTOR in alleviating MIRI needs further study.

### Potential compounds for the treatment of MIRI

Considering the important role of inhibiting ferroptosis and regulating mitophagy in alleviating MIRI, we have summarized the potential drugs that can inhibit ferroptosis and/or regulate mitophagy (Table 1). In pathological conditions, since mitochondria are not the only ROS source, theoretically compounds that can both regulate mitophagy and inhibit ferroptosis should have better medicinal potential than compounds that inhibit only ferroptosis or regulate mitophagy alone. But it is a pity that although there are many compounds that can alleviate MIRI by targeting mitochondrial ROS clearance (Peng et al., 2022), there are few natural compounds have been reported to alleviate MIRI by regulating mitophagy or inhibiting ferroptosis. This indicates that although ferroptosis inhibition and mitophagy regulation play important roles in MIRI relief from a mechanism perspective, the study of active compounds for the treatment of MIRI based on ferroptosis and mitophagy needs to be further studied. Berberine alleviates MIRI through HIF-1 *α*/BNIP3 pathway (Zhu et al., 2020), and pentauterine B alleviates MIRI by inhibiting phosphorylated mTOR (Lu et al., 2019), providing direct evidence for the important role of mTOR and HIF in regulating mitophagy in MIRI alleviation. Therefore, referring to our previous analysis, we believe that HIF-1, mTOR and NLRP3 may become important targets for screening effective drugs to treat MIRI, based on the simultaneous regulation of mitophagy and ferroptosis.

**Table 1:** The potential durgs that can inhibit ferroptosis and/or regulate mitophagy.

Compounds	Component types	Model	Effect	Target/ mechanism	Ref
Aringenin	Flavonoid	Rat model of MIRI.	Inhibition of ferroptosis	Regulating Nrf2/System Xc-/ GPX4 axis	Xu et al. (2021b)
Cyanidin-3-Glucoside	Flavonoid	1. Rat model of MIRI.	Inhibition of ferroptosis	Downregulating LC3II/LC3I, reducing autophagosome number, downregulating TfR1 expression, and upregulating the expressions of ferritin heavy chain 1 and GPX4	Shan et al. (2021)
		2. Oxygen-glucose deprivation/reoxygenation (OGD/R) model of H9C2 cell.			
Icariin	Flavonoid	OGD/R model of H9C2 cell	Inhibition of ferroptosis	Activating the Nrf2/HO-1 signaling pathway;decreasing content of Fe^2+^ and increasing expression of GPX4	Liu et al. (2021b)
Xanthohumol	Flavonoid	1. Ferroptosis model of H9C2 cell.	Inhibition of ferroptosis	Decreasing the ROS and LPO, chelating iron, reducing the NRF2 protein level, and modulating the protein level of GPX4.	Lin et al. (2022)
		2. Rat MIRI model with Langendorff Heart Perfusion System *In vitro*.			
Resveratrol	Polyphenol	OGD/R model of H9C2 cell.	Inhibition of ferroptosis	Reducing Fe^2+^ content, decreasing TfR1 expression, and increasing the expressions of ferritin heavy chain 1 and GPX4	Li et al. (2022b)
Histochrome	A water-soluble form	Rat model of MIRI.	Inhibition of ferroptosis	Upregulating the expression of nuclear factor erythroid 2-related factor (*Nrf2*) and its downstream genes, maintaining the intracellular glutathione level, upregulating the activity of GPX 4	Hwang et al. (2021)
Gossypol Acetic Acid	Acetic Acid	1. Ferroptosis model of H9C2 cell.	Inhibition of ferroptosis	Reducing lipid peroxidation, decreasing the protein levels of ACSL4 and NRF2, and increasing the protein levels of GPX4.	Lin et al. (2021)
		2. Rat heart MIRI model established by Langendorff Heart Perfusion System.			
Ferulic acid	Polyphenol	Rat model of MIRI.	Inhibition of ferroptosis	Reversing the increased level of the Ptgs2 mRNA, Fe^2+^ accumulation, and a decreased GSH/GSSG ratio caused by ferroptosis. Upregulation of AMPK *α*2 and GPX4 expression	Liu et al. (2021c)
Britanin	Lactone	1. Rat model of MIRI.	Inhibition of ferroptosis	Upregulating GPX4 through activation of the AMPK/GSK3b/Nrf2 signaling pathway	Lu et al. (2022)
		2. OGD/R model of H9C2 cell.			
Baicalin	Flavonoid glycoside	1. Rat model of MIRI	Inhibition of ferroptosis	Reverse ferroptosis induced lipid peroxidation, iron accumulation, and activated TfR1 signal and nuclear receptor coactivator 4 (NCOA4)-medicated ferritinophagy	Fan et al. (2021)
		2. OGD/R model of H9C2 cell.			
Berberine	Alkaloid	1. Rat model of MIRI	Promoting mitophagy	Mediating HIF-1 *α*/BNIP3 pathway	Zhu et al. (2020)
		2. OGD/R model of H9C2 cell.			
Gerontoxanthone I and Macluraxanthone, Xanthone	Xanthone	OGD/R model of H9C2 cell.	Promoting mitophagy	Mediating PINK1/Parkin pathway	Xiang et al. (2020)
Panax Notoginseng Saponins	Saponins	Rat model of MIRI	Promoting mitophagy	Mediating HIF-1a/BNIP3 pathway	Liu et al. (2019b)
Carvacrol	Monoterpene phenol	1. Rat model of MIRI	Promoting mitophagy	Mediating PINK1/Parkin pathway	Yan, Yang & Cheng (2021)
		2. OGD/R model of H9C2 cell.			
AstragalosideIV and Ginsenoside Rg1	Triterpenoid saponin	Rat model of MIRI	Inhibition of excessive mitophagy	Mediating PINK1/Parkin pathway;upregulating expression of HIF- *α* and downregulating expression of NRF-1	Zhang et al. (2020c)
Schisandrin B	lignan	Mice model of MIRI	Promoting mitophagy	Increasing HIF-1 *α* and Beclin1 protein expression, inhibits the expression of phosphorylated mTOR	Lu et al. (2019)
Salvianolic acid B	Phenolic acid	OGD/R model of H9C2 cell.	Inhibition of mitophagy	Decreasing LC3-II/LC3 ratio and expression of Nix	Xin et al. (2020)
Tongxinluo Capsule	Chinese herbal	Rat model of MIRI	Promoting mitophagy	Activating PINK1/Parkin Pathway	Yang et al. (2021)
Shenmai Injection	Chinese herbal	Rat model of MIRI	Inhibition of ferroptosis	Mediating Nrf2/GPX4 signaling pathway	Mei et al. (2022)
Luhong Formula	Chinese herbal	Rat model of MIRI	Inhibition of ferroptosis	Mediating SLC7A11/GPX4 signaling pathway	Cai et al. (2022)

## Conclusions and Perspective

In present, although there are many studies on the mechanism of ferroptosis and mitophagy, the current drug studies on the treatment of MIRI mainly focus on inhibiting ferroptosis or regulating mitophagy alone. Damaged mitochondria will produce a large number of ROS, which promotes ferroptosis. Appropriate mitophagy can reduce mitochondria ROS production to alleviate MIRI. Our analysis showed that ferroptosis in MIRI may be mitochondrial ROS dependent. Since mitochondria are not the only source of ROS in cells, reducing ROS by regulating mitophagy can alleviate but not completely block ferroptosis. Therefore, compounds can simultaneously regulate mitophagy and inhibit ferroptosis have great potential to treat MIRI. Act as the “link” between ferroptosis and mitophagy, HIF, NLRP3 and mTOR can regulate both mitophagy and ferroptosis. Therefore, we analyzed the relationship between ferroptosis and mitophagy in MIRI based on the role of HIF, mTOR and NLRP3, summarized potential drugs that could treat MIRI by regulating mitophagy and/or ferroptosis, hoping to provide reference for the drug and methods development of MIRI therapy.

## Additional Information and Declarations

### Data Availability

The following information was supplied regarding data availability: This article is a literature review and there is no raw data.
